# Sensitive and Specific Analyses of Colorectal Cancer Recurrence through Multiplex superRCA Mutation Detection in Blood Plasma

**DOI:** 10.3390/cancers16030549

**Published:** 2024-01-27

**Authors:** Emma Sandberg, Luís Nunes, Per-Henrik Edqvist, Lucy Mathot, Lei Chen, Tomas Edgren, Shahed Al Nassralla, Bengt Glimelius, Ulf Landegren, Tobias Sjöblom

**Affiliations:** 1 Science for Life Laboratory, Department of Immunology, Genetics and Pathology, Uppsala University, SE-751 85 Uppsala, Sweden; emma.sandberg@igp.uu.se (E.S.); luis.nunes@igp.uu.se (L.N.); per-henrik.edqvist@igp.uu.se (P.-H.E.); lucy.mathot@igp.uu.se (L.M.); lei.chen@raritybioscience.com (L.C.); shahed.alnassralla.9832@student.uu.se (S.A.N.); bengt.glimelius@igp.uu.se (B.G.); 2 Rarity Bioscience AB, SE-752 37 Uppsala, Sweden; tomas.edgren@raritybioscience.com

**Keywords:** colorectal cancer, recurrence, cfDNA, ctDNA

## Abstract

**Simple Summary:**

Analysis of mutant DNA, leaking from tumors into blood plasma, can be used to detect tumor recurrence. We demonstrate that a novel, multiplex method termed superRCA is useful to analyze plasma for somatic hotspot mutations prevalent in solid cancer genomes to detect the recurrence of colorectal cancer.

**Abstract:**

Mutation analysis of circulating tumor DNA (ctDNA) has applications in monitoring of colorectal cancer (CRC) patients for recurrence. Considering the low tumor fraction of ctDNA in cell-free DNA (cfDNA) isolated from blood plasma, the sensitivity of the detection method is important. Here, plasma DNA collected at diagnosis and follow-up from 25 CRC patients was analyzed using a multiplex superRCA mutation detection assay. The assay was also performed on genomic DNA (gDNA) from tumor and normal tissue from 20 of these patients. The lower limit of detection for most sequence variants was in the range of 10^−5^, while when analyzing cfDNA from plasma with a typical input of 33 ng, the practical detection limit was ~10^−4^ or 0.01% mutant allele frequency (MAF). In 17 of 19 patients with identified hotspot mutations in tumor gDNA, at least one hotspot mutation could be detected in plasma DNA at the time of diagnosis. The MAF increased at subsequent time points in four of the patients who experienced a clinical relapse. Multiplex superRCA analysis of the remaining six patients did not reveal any hotspot mutations. In conclusion, multiplex superRCA assays proved suitable for monitoring CRC patients by analyzing hotspot mutations in cfDNA, and dynamic changes in MAF were observed in patients with clinical relapse.

## 1. Introduction

Colorectal cancer (CRC) is the third most common cancer type globally, and the second most common cause of cancer death [1]. The five-year recurrence rate in a Swedish population-based cohort from 2018 was 5% in stage I, 12% in stage II, and 33% in stage III colon cancer [2]. It is important to assess the risk of recurrent disease, and to promptly detect signs of recurrence, as well as risks for toxicity and expected benefits of adjuvant treatment in deciding when to recommend chemotherapy and what type of chemotherapy to use. In current guidelines, adjuvant chemotherapy is recommended for patients with stage II colon cancer with high-risk criteria, and for all with stage III. Currently, the risk of recurrence is assessed by the tumor-node-metastasis (TNM) stage, microsatellite instability/mismatch repair (MSI/MMR) status, number of lymph nodes, and additional clinicopathological characteristics, such as perineural, vascular or lymphatic invasion, histological grade and subtype, tumor obstruction, and levels of carcinoembryonic antigen (CEA) [3]. Biomarkers can be of benefit for assessing the risk of recurrence, including the Immunoscore^®^, circulating tumor DNA (ctDNA), and gene signatures [4,5]. The Immunoscore^®^ test was predictive of time to recurrence in a large prospective cohort of patients with TNM stages I–III, although its potential role in predicting benefits from chemotherapy in stage II colon cancer patients remains to be assessed [4]. Postoperative measurement of cell-free DNA (cfDNA) for tumor-specific mutations is showing promising results for identifying individuals with a high risk of recurrence [5]. 

The phenomenon that tumors shed material into the blood stream was discovered a few decades ago [6,7]. This knowledge has given rise to the field of liquid biopsy, i.e., the opportunity to investigate disease anywhere in the body by analyzing markers released to the blood stream, such as circulating tumor cells, ctDNA, exosomes, and tumor-educated platelets [8,9]. One of the most promising classes of liquid biopsy biomarkers for monitoring solid tumors is ctDNA, the levels of which strongly correlate with tumor volume and with the presence of liver metastasis [10]. The utility of ctDNA to predict the recurrence of CRC is currently being studied in several clinical trials [11,12]. In a prospective study of 230 patients with resected stage II colon cancer, those who were ctDNA-positive postoperatively had a higher risk of recurrence than those with negative ctDNA. This was true whether or not patients received adjuvant chemotherapy [5]. By using ctDNA for treatment guidance in stage II colon cancer, the use of adjuvant chemotherapy was reduced without compromising recurrence-free survival [13]. In stage III colon cancer patients, ctDNA detected postoperatively was associated with shorter recurrence-free survival. However, among stage III postoperative patients, only 42% of recurrences were detected using ctDNA analysis [14]. Most patients diagnosed with metastatic CRC have detectable ctDNA [15]. In a prospective study, the presence of ctDNA in post treatment samples from 35 metastatic CRC patients was associated with recurrence [16]. Detection of measurable residual disease (MRD) via ctDNA after curative surgery in earlier stages requires more sensitive technology, compared to monitoring of metastatic disease due to the generally low amount of ctDNA in plasma samples [17]. 

There is considerable shedding of tumor fragments in CRC, which makes the disease suitable for liquid biopsy [18]. Considering that the ctDNA/cfDNA ratio can be much lower than 1%, sensitive detection methods are warranted. Analyses of ctDNA can be conducted with both PCR-based techniques and through next-generation sequencing (NGS). Although NGS provides a potential to retain whole-genome sequencing data, in clinical practice, it is usually limited to panels of hotspots of tens up to a few hundred genes. PCR-based methods can provide a higher level of sensitivity (mutant allele frequencies [MAF] for detection ≤ 0.01%), but on the downside, the number of detectable mutations is limited. 

superRCA is an ultrasensitive method for detecting DNA sequence variants from malignant cells in up to a 100,000-fold excess of DNA from normal cells in peripheral blood or bone marrow. The ability of superRCA to reveal very low frequencies of mutations is a consequence of two factors. First, mutant and wild-type sequences are distinguished in accurate ligase-mediated padlock probe ligation reactions, but a low proportion of mistaken distinction between the sequence variants is to be expected. However, since this padlock probing reaction is applied to an RCA product containing hundreds of copies of the target sequence, the occasional incorrect reaction by a padlock probe is invisible against the vast majority of correctly genotyped copies of the target sequences. Second, in the superRCA procedure, around one million products are evaluated, which is sufficient to record mutant variants at frequencies of one in 100,000 or lower, and allows precise counts of mutants occurring at higher frequencies. The superRCA method was previously used to monitor patients with leukemia in order to detect early recurrence [19]. When comparing superRCA to clinically used NGS and droplet digital PCR (ddPCR) assays, superRCA is capable of detecting more mutations and lower frequencies than ddPCR, and the method is less costly and has a shorter turn-around time than NGS [19]. 

Here, we have evaluated the performance of a superRCA assay for detecting the recurrence of solid tumors by analyzing cfDNA from CRC patients. 

## 2. Materials and Methods

### 2.1. Tumor Tissue and Plasma Samples

Tumor tissue and plasma samples were obtained through the Uppsala-Umeå Comprehensive Cancer Consortium (U-CAN) project (www.u-can.uu.se, accessed on 11 July 2023) [20]. The study included 74 plasma samples from 25 CRC patients from the population-based, prospective longitudinal biobank U-CAN (Table 1) [20]. Tumor hotspot mutations in *BRAF*, *KRAS*, *NRAS*, and *PIK3CA* were derived from molecular pathology reports in the patients’ medical records. Control genomic DNA (gDNA) was purified from normal tissue, and gDNA was extracted from primary tumor tissue after surgery for samples from 20 of these patients. A volume of 1.8 mL plasma isolated from blood drawn at the time of diagnosis and at two later time points was obtained from all patients, except for patient UU035, where only two samples in total were included. Postoperative plasma samples were available from seven patients. UU004 did not have surgery, and hence, for this patient, the sample collected week six from diagnosis was used instead, but included in the postoperative samples. The rest of the samples were collected during the course of the disease, with a time frame of 16 weeks up to four years (Table 2).

### 2.2. DNA Preparation

Control tumor gDNA was extracted from fresh-frozen sections using the NucleoSpin Tissue kit (Macherey-Nagel, Hilden, Germany), while matched normal gDNA was extracted from blood samples using the Nucleospin 96 Blood core kit (Macherey-Nagel, Hilden, Germany). Control normal and tumor gDNA samples were available as purified gDNA with high concentrations (7.8–40 ng/μL) in 30–100 μL aliquots. Plasma samples were received as 8 aliquots of 220 μL each. The 8 aliquots from each plasma sample were pooled and the cfDNA was purified using the QIAamp Circulating Nucleic Acid kit (Qiagen cat.55114, Hilden, Germany) and a QIAvac 24 Plus (Qiagen cat.19413, Hilden, Germany) according to the manufacturer’s recommendations and eluted in a total volume of 30 μL elution buffer. The cfDNA concentrations were determined using the Qubit dsDNA HS Assay Kit (Thermo Fisher Scientific, Waltham, MA, USA).

### 2.3. superRCA Assay Principle

The MAF of the samples were determined with the multiplex CRC superRCA Mutation Kit (Rarity Bioscience, Uppsala, Sweden) using a CytoFLEX flow-cytometer (Beckman Coulter, Brea, CA, USA) for read-out. The multiplex CRC superRCA Mutation Detection assay is based on and operated under the same conditions as the original superRCA protocol [19]. Target specific reagents were provided by the manufacturer. superRCA is a single-tube, targeted multiplex genotyping assay that combines two rounds of rolling circle amplification (RCA) with sequence-specific genotyping padlock probes. First, the purified DNA sample undergoes a limited number of cycles of multiplex PCR to enrich sequences of interest. Next, the 3′ and 5′ ends of one of the strands of amplification products are allowed to hybridize next to each other to a complementary oligonucleotide, allowing the ends of the strands to be joined by ligation to form circular DNA molecules. The circular strands then template RCA reactions that yield, for each starting DNA circle, one linear single-stranded concatemer with several hundred repeats of the original amplicon. Next, genotyping padlock probes, specific for the mutant or wild-type sequences, are introduced to interrogate the repeated targets in individual RCA products. The assay is tolerant for the occasional mistyping by the padlock probes, since according to the majority vote principle, the most abundant signal coming from each genotyped RCA product accurately reflects the true genotype of that starting DNA circle. The circularized genotyping padlock products, linked to RCA products, are then, in turn, replicated in secondary RCA reactions. The products of the secondary RCA reactions remain anchored to the first RCA products by hybridization. Each of the starting RCA products result in individual brightly fluorescent clusters of DNA by hybridizing fluorescent oligonucleotides, differentially labeled for detecting products of mutant or wild-type sequences.

Each superRCA reaction yields ~10^6^ products that can be distinguished and counted using standard flow cytometry. The highly accurate genotyping of the repeated sequence of the first RCA products, along with the large number of superRCA products that are counted, together, account for the very sensitive and precise detection of mutant sequences—suitable for detecting MRD in patient plasma samples. The superRCA assay is quantitative in the sense that it gives a ratiometric result of the amount of mutated vs. wild-type events. All of these variants have their respective wild-type and mutant sequence on the same position and hence are located on the same amplicon, so the assay is not directly affected by amplification efficiency as the ratio will remain intact. The assays are, however, designed to provide a similar amount of events with minimum levels for acceptability, and there is a cycle guide depending on the amount of input.

### 2.4. Pre-Amplification

The 10 DNA amplicons encompassing 44 mutations covered by the multiplex CRC superRCA Mutation Kit (Rarity Bioscience, Uppsala, Sweden) (Table S1) were enriched in the samples by multiplex PCR using the CRC Pre-amplification buffer, supplemented with Pre-amp Enzyme 1 and 2 supplied with the kit. Pre-amplification reactions were performed in 0.2 mL 96-well PCR plates (Nest Biotechnology, Wuxi, China) in a final volume of 50 μL, using a SimpliAmp Thermal Cycler (Thermo Fisher Scientific, Waltham, MA, USA). The program started with a 10 min incubation at 37 °C, followed by 30 s denaturation at 98 °C before cycling between 30 s at 98 °C and 120 s at 62 °C. The program ended with a 5 min extension at 72 °C. 

The number of cycles were adjusted according to the total DNA input in the PCR sample, and also the integrity of the DNA as cfDNA in plasma was fragmented. Plasma samples with >25 ng total cfDNA were amplified in 14 cycles, while 16 PCR cycles were used to amplify plasma samples with <25 ng total cfDNA. The whole 30 μL DNA sample from plasma cfDNA purification was used in the final superRCA analysis, except for the UU035 follow-up sample where 4.4 μL, corresponding to 33 ng DNA, was used. A total of 33 ng from each of the control gDNA samples with identified hotspot mutations was analyzed. The assay was performed according to manufacturer´s instructions (Rarity Bioscience, Uppsala, Sweden).

### 2.5. superRCA Reactions

All superRCA reactions were performed in a full-skirted 0.1 mL 96-well PCR plate (Nest Biotechnology, Wuxi, China) using an automated protocol on an OT-2 Pipetting Robot fitted with a GEN2 Thermocycler Module (Opentrons, Long Island City, NY, USA) using the buffers supplied with the kit according to the manufacturer’s recommendations. Of the PCR enriched samples, 1.2 μL were added to 20 μL PCR Clean-Up Buffer, supplemented with 1st Clean-up Enzyme and incubated at 37 °C for 5 min. Next, 5 μL Clean-Up Buffer supplemented with 2nd Clean-Up Enzyme was added and incubated at 37 °C for 10 min, followed by 55 °C for 5 min. Next, 5 μL Ligation Buffer supplemented with DNA ligase was then added and the mixture was incubated at 95 °C for 2 min, followed by 55 °C for 15 min. After ligation, 5 μL RCA buffer supplemented with RCA polymerase was added and incubated at 37 °C for 25 min, followed by 60 °C for 10 min. Next, 5 μL of a genotyping solution with the appropriate Genotyping padlock probes was added and incubated at 55 °C for 30 min. This was followed by addition of 5 μL Clean-Up Buffer, supplemented with 3rd Clean-Up Enzyme and incubated at 37 °C for 15 min, 75 °C for 15 min. Next, 5 μL RCA buffer supplemented with superRCA polymerase was added and the mixture was incubated at 37 °C for 25 min. The final superRCA products were labeled by addition of fluorescent hybridization oligonucleotides in 5 μL Labeling buffer and incubated at 45 °C for 15 min.

### 2.6. Flow Cytometry

The final superRCA reaction products were identified and enumerated using a CytoFLEX flow-cytometer (Beckman Coulter, Brea, CA, USA). The superRCA products derived from wild-type alleles were labelled with FITC and those derived from mutant variants were labelled with Cy-5. Acquisition was set at 30 μL/min for 75 s. The number of events depended on the amount of input but were in the range 400,000–1,000,000. Negative controls (wild-type control) were included at all times when the assay was performed and positive controls were included when available. 

### 2.7. Ethical Approval

The patients gave written informed consent to donate biopsy and blood samples for research when they were included in the U-CAN cohort. The ethical permit EPN Uppsala 2015–419 regulated the use of the samples and clinical data from the patients in this project. 

## 3. Results

### 3.1. Patient Cohort and cfDNA Purification

To evaluate the performance of the superRCA assay for recurrence detection, blood samples were collected at diagnosis, postoperatively and/or during follow-up from 25 patients along with tissue samples from 20 of these patients (Table 3). Most (60%) were below 75 years old, and male (60%). Three patients (12%) were in stage I, five (20%) in stage II, 15 (60%) in stage III, and two (8%) were in stage IV. The majority had a primary tumor located in the colon (72%). Both patients that experienced clinical relapse after treatment or continued recurrence-free were included. 

The study included gDNA and plasma samples both from patients with clinically identified and unknown genetic tumor markers (Table 1). Control gDNA isolated from tumor and matching normal tissue biopsies were obtained from 20 patients. Fifteen of the gDNA patient samples had one or two identified hotspot mutations in the four routinely analyzed genes, while the remaining five had wild-type hotspot backgrounds. In addition, plasma samples were collected at the time of diagnosis and at later time points from these 20 patients and from five additional patients (Table 2 and Figure S1). The five additional patients included four with identified hotspot mutations and one wild-type (Table 1). In total, 12 unique hotspot mutations in four different genes (*KRAS*, *PIK3CA*, *BRAF*, and *NRAS*) were included in six different amplicons that were amplified jointly (KRAS146, KRAS12, PIK3CA545, PIK3CA1047, BRAF600, and NRAS12). Fifteen patients had a single hotspot mutation among the targeted positions, four had two hotspot mutations, and six had none in the target genes (Table 1).

As expected, the amount of cfDNA varied in the different plasma samples from a total of 7.13 ng cfDNA recovered from the UU019 follow-up sample to 222.9 ng cfDNA recovered from the UU035 follow-up sample. The average cfDNA amount recovered was 22.43 ng, 15.91 ng, and 26.62 ng at diagnosis, postoperatively, and during follow-up, respectively (Table 1 and Figure S2). 

### 3.2. superRCA Analysis of Samples with Identified Hotspot Mutations

**Figure 1:**
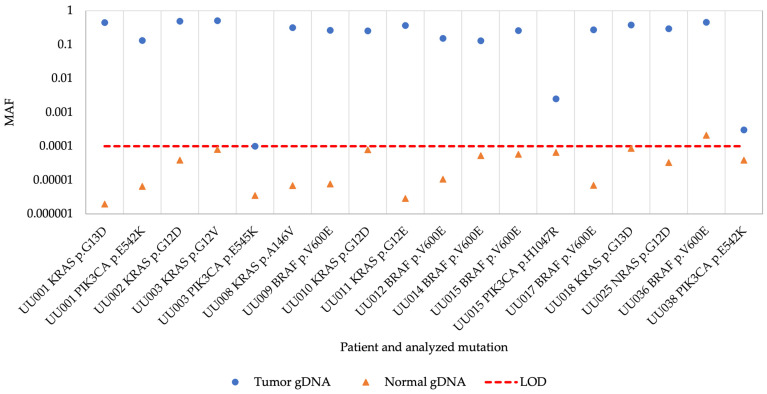
Mutant allele frequency of tumor and normal gDNA samples. MAF, mutant allele frequency; gDNA, genomic DNA; LOD, limit of detection.

All previously identified hotspot mutations were detected in the tumor biopsy gDNA samples using the superRCA mutation assay although the MAF was low in three biopsies (Figure 1 and Table S2). All corresponding control gDNA samples from normal tissue were negative except for a sample from patient UU036 with a *BRAF* V600E mutation, where the mutation was detected at a MAF of 0.0002 (0.02% MAF) in the normal control sample. These results demonstrate that the assay efficiently detected different hotspot mutations in the gDNA control samples.

**Figure 2:**
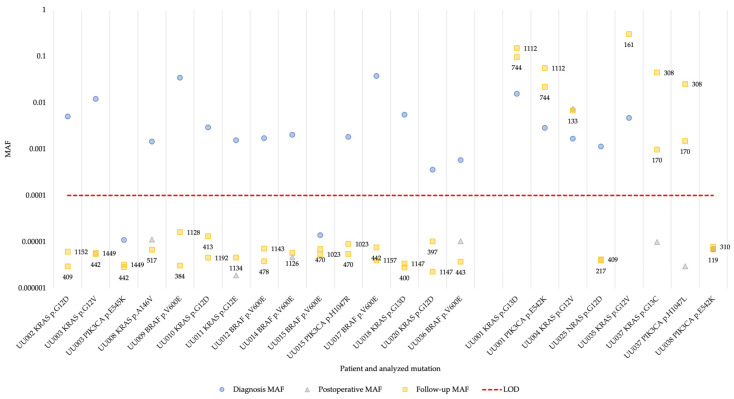
Changes in MAF determined by superRCA in plasma cfDNA for 19 patients over the course of their disease. The patients are grouped by non-recurrent vs. recurrent disease, with non-recurrent to the left and recurrent to the right. The numbers next to the squares are days from surgery to follow-up sample time point. MAF, mutant allele frequency; LOD, limit of detection.

Next, we analyzed the cfDNA isolated from plasma from the 19 patients with identified hotspot mutations. In 17 of the patients (94% of patients with available diagnosis plasma sample), at least one of the hotspot mutations present in the tumor could be detected in cfDNA at the time of diagnosis (Figure 2 and Table S3). One of the patients (UU038) had a MAF below the detection limit of 0.0001 (0.01% MAF) in plasma collected at diagnosis. In the postoperative samples, one of the patients were positive and had increased MAF for the identified hotspot mutations, while the remaining patients showed background levels of mutant alleles in the cfDNA (Figure 2). This pattern persisted in the follow-up samples where the MAF of the patient positive for the hotspot mutations remained increased. The MAF of three patients (UU001, UU035, and UU037) was increased during follow-up, while measurements for the rest of the patients remained at background levels (Figure 2). For one patient whose cfDNA sample taken 20 days postoperatively, the sample was negative (UU037) and both identified hotspot mutations were detected in the cfDNA samples collected during follow-up.

Comparing the plasma cfDNA superRCA results with patient data revealed that the four patients with detectable hotspot mutations in cfDNA after surgery (UU001, UU004, UU035, and UU037) indeed experienced clinical relapse. An additional patient (UU025) also experienced clinical relapse after surgery, but the *NRAS* p.G12D mutation was not detected in the follow-up plasma samples. It is possible that a tumor cell clone lacking the *NRAS* p.G12D mutation was responsible for the relapse in this patient. The recurrent tumor was not genotyped and the malignant cells carrying the *NRAS* p.G12D mutation could then be cleared during surgery or adjuvant chemotherapy. Moreover, patient UU038 also experienced clinical relapse, but the *PIK3CA* p.E542K mutation was not detected in any sample, neither at diagnosis nor at follow-up. The remaining 13 patients exhibiting background levels of hotspot mutations remained recurrence-free after surgery (Table 1 and Table 2, Figure 2 and Figure S1).

### 3.3. Multiplex superRCA Analysis of Wild-Type Samples

One of the advantages of the superRCA mutation detection assay is the possibility to analyze multiple mutations in limited cfDNA samples. The unbiased, low-cycle, multiplex, pre-amplification in the superRCA procedure enriches the relevant DNA sequences in samples without affecting the MAF. Thus, after the pre-amplification, the samples can be divided and analyzed in parallel in the 96-well format for any mutation present in the amplicons included in the panel. Amongst the patients without *RAS*, *BRAF*, and *PIK3CA* mutations (i.e., the wild-type of these genes), three experienced clinical relapses after surgery (UU005, UU006, and UU007) (Figure S1). Since the recurrent tumors were not genotyped and the genetic signature was thus unknown, we proceeded to analyze cfDNA in the last samples collected from these patients and from patient UU025, discussed above, for all 44 mutations included in the superRCA CRC Mutation assay target panel (Table S1). The last follow-up sample for patient UU007 was unfortunately lost during cfDNA purification, so instead, the earlier follow-up plasma cfDNA was analyzed for this patient. However, the earlier follow-up sample for patient UU007 was collected 750 days prior to recurrent disease. The rationale was that in the relapse patients with wild-type tumors, additional mutations might have been acquired or selected for during treatment. Each of the 44 wild-type genotypes encoded in the 10 different amplicons were analyzed in the samples, but none of the samples were positive for any of the mutations included in the panel. 

## 4. Discussion

Operated cancer patients increasingly have their tumors sequenced to identify patient-specific, sometimes actionable, mutations. This also provides an opportunity to search for the identified mutations in blood liquid biopsies. Serial analyses of cfDNA have proven helpful in several studies to provide an estimate of the recurrence risk and, if repeated samples are taken, to detect recurrence prior to any clinical or radiological manifestation of relapse [21,22]. According to the Swedish national guidelines from 2016 and 2020 for management of CRC patients, computed tomography of the chest and abdomen and measurement of CEA are recommended after one and three years to detect the recurrence of radically resected colon cancer [23]. This internationally low intensity of follow-up is based upon a large multicenter study (COLOFOL) where more intense follow-up did not result in better survival [24]. However, even if CEA has a sensitivity of less than 70% for detecting recurrence and imaging cannot detect small recurrences early, these follow-up methods are internationally accepted at much higher frequencies. In a recent study, cfDNA testing could detect recurrences in a median of 8.7 months before imaging [25]. In yet another study, analysis of cfDNA revealed recurrence 9.4 months before recurrence was detected by imaging [22]. Hence, screening for ctDNA appears to offer greater sensitivity for recurrence than CEA and imaging in clinical practice. However, measurement of ctDNA is not yet routine outside highly specialized centers. 

The cfDNA analysis can be tumor-informed or tumor-agnostic. Tumor-agnostic approaches for ctDNA detection have been suffering from low sensitivity due to the pre-defined list of targets in the assay aiming for assay robustness and cost effectiveness. The tumor-informed approach has better sensitivity due to the design of personalized mutation assays, but presupposes mutational analysis of tumor tissue. However, the sensitivity of the tumor-agnostic approach has been improved by adding DNA methylation as epigenetic markers [26]. Tumor-agnostic cfDNA analyses can allow for earlier assessment of MRD status, as they do not depend on tissue retrieval for sequencing and customization of a ctDNA panel, hence minimizing delays in starting of adjuvant therapy [27]. The analysis of cfDNA without prior knowledge about the mutation status may also prove applicable to screening for previously unknown malignant disease. Using Safe SeqS assays to assess a single tumor-specific somatic mutation in cfDNA in each patient, 42% of recurrences were detected [14]. Another tumor-informed, hybrid-capture cfDNA sequencing MRD assay called AlphaLiquid^®^ Detect was used to target large sets of patient-specific mutations by sequencing, efficiently predicting the recurrence of disease [28]. Two blood-based liquid biopsy tests have been approved by the Food and Drug Administration (FDA) for guidance in treatment decisions for patients with solid cancer. Guardant360 CDx and FoundationOne Liquid CDx were both approved in 2020 with the intention to identify cancer patients who may benefit from certain treatments by detecting genetic alterations in cfDNA in plasma [29,30]. 

In this study, we evaluated a tumor-informed mutation detection assay, superRCA. The assay was applied to samples from CRC patients in stages I through IV. At least one hotspot mutation could be detected in plasma cfDNA at diagnosis in 17 of 19 patients with identified hotspot mutations in tumor gDNA, and the assay revealed dynamic changes in MAF over time. In four of these patients, the MAF increased postoperatively and/or during follow-up, which accorded with their recurrent clinical course. Hotspot mutations were not detected for two of the patients who experienced recurrent disease. There might be several causes for this, one being tumor heterogeneity. Due to intratumoral heterogeneity caused by dynamic changes occurring over time in the tumor and the uneven distribution of subpopulations of mutation clones across the tumor tissue, hotspot mutations might have been missed [31]. The levels of DNA released to the blood stream from the tumor or from subclones therein are likely to vary over time so that a given sample may contain undetectable amounts of the analyzed mutations. Another possible reason is the limited mutation panel. Since this study based the MRD assessment on tissue data, mutations outside of the current routinely used panel would have been missed. However, even though the mutation panel is small, it is clinically relevant since it includes the actionable mutations currently checked before treatment decisions. The panel can easily be expanded to include further mutations as needed. It is also possible to develop customized panels to ensure that more mutations are being monitored in samples from a given patient.

None of the wild-type samples were positive for any of the mutations included in the superRCA CRC Mutation assay target panel. In this case, none of the investigated recurrences seem to have acquired any of the mutations covered by the mutation target panel. Even though these samples were negative for all the mutations tested, analyzing 44 different mutant variants in cfDNA plasma samples with limited DNA amounts demonstrated the potential of the assay for high throughput screening for numerous mutant variants in scarce DNA samples. Blind screening of the cfDNA samples collected at diagnosis using the whole superRCA CRC Mutation target panel assay would correctly genotype 17 out of 25 (68%) patients in this cohort. In a prospective study with metastatic CRC patients, ctDNA levels were analyzed in post-treatment and follow-up blood samples. A total of 5 out of 35 patients were ctDNA-positive for a *KRAS*, *NRAS*, or *BRAF* mutation by ddPCR, and in total, 17 patients had recurrent disease [16]. In comparison to our study, Boysen et al. [16] reported more cases and more recurrences. However, slightly fewer recurrences were detected by their methods, 4 out of 10 (40%), compared to 4 out of 6 (67%) by cfDNA analysis using superRCA. This comparison only includes the patients with known mutational status. Moreover, no false positive cases were detected by superRCA, while one was observed by Boysen et al. [16]. Regarding the false-negative results, the rationale was that in the recurrences among patients with wild-type tumors, additional mutations might have been acquired or selected for during treatment. For the patients with known mutational status with false-negative results the same logic applies.

A delay of at least two to four weeks after curative surgery is required in MRD assessment through cfDNA testing, in order to ensure good sensitivity. The presence of high levels of normal cfDNA after surgery can dilute the ctDNA levels leading to lower sensitivity [18,32]. The timing of blood sampling affects the ctDNA levels since the release of ctDNA can be affected by factors such as the surgery and inflammatory or infectious processes. Thus, depending on the scientific question, the blood sampling should be planned accordingly. During treatment, both normal and tumor cells are directly affected, influencing ctDNA levels, possibly with a detectable change within days to weeks. Long-term changes may occur in weeks to months and correlates more accurately to actual shrinkage of the tumor [33]. In this study, time points for the included samples varied. The time points for the included plasma samples were limited by the availability of plasma samples with sufficient amount. This could be addressed by collecting the samples at predetermined time points with regard to patient-specific treatments, as is done in U-CAN, while securing sufficient amounts of each plasma sample. With shorter intervals between sample collection, it will be possible to investigate how long before clinically manifest recurrent disease that hotspot mutations can be detected in cfDNA. 

The superRCA technique described here, presents several advantages in monitoring cancer patients by screening for patient-specific mutations compared to alternative approaches. While rare mutation monitoring by sequencing is highly flexible, it is costly to achieve high sensitivity, and the turn-around time is long. superRCA can be performed in a few hours, and is capable of detecting mutations at rates as low as 1 in 100,000 [19]. Moreover, as shown here, several mutations can be targeted in parallel in the limited DNA samples available by liquid biopsy, and a read-out only depends on an installed base of flow cytometers. The superRCA procedure requires a series of additions of reagents and incubations over a few hours total reaction time, but these steps have been automated using a simple lab robot. An obvious limitation of the superRCA technique compared to DNA sequencing is that it can only distinguish specific nucleotide variants, whereas sequencing can reveal previously unknown variants. 

The ability of superRCA to reveal the recurrence of solid tumors should be validated in larger cohorts in future studies, as well as in prospective studies with predetermined time points for sample collection to be able to draw disease relevant conclusions. For future studies, superRCA should be compared to current guidelines as well as alternative technologies for ctDNA analysis. Moreover, it will be necessary to evaluate in future studies the potential of superRCA to detect recurrence at earlier time points than what is possible using the current clinical practice with CEA levels and imaging. Since the MRD assessment was based on tissue data, relevant mutations outside the current panel would have been missed, but could be targeted in future studies. Patient-specific mutations identified by initial sequencing upon diagnosis can be used to establish dedicated assays that complement the standardized multiplex panel used in this study. The sensitivity and multiplexing potential of superRCA for mutation analysis in cfDNA may allow earlier detection of recurrent disease for prompt therapy adjustment. By predicting the risk of micrometastatic disease, the assays may inform selection of adjuvant chemotherapy in stage II colon cancer patients and of optimal combination and duration of treatment in stage III colon cancers. 

## 5. Conclusions

The superRCA assay performed well in analyses of hotspot mutations in cfDNA. The assay is a promising tool in monitoring CRC patients for recurrence via ctDNA. The analyses could potentially decrease recurrence risks by allowing treatment to be promptly initiated for those in greatest need. Further validation of the assay in larger cohorts will be required. 

## Figures and Tables

**Table 1:** Colorectal cancer patients included in the study. In total, plasma samples of 1.8 mL each were obtained from 25 CRC patients at diagnosis, postoperatively and/or during follow-up. Genomic DNA (gDNA) from tumor and normal tissue were available from 20 of the patients. Wild-type indicates that no mutations were identified for the hotspot mutations. gDNA, genomic DNA; cfDNA, cell-free DNA; N/A, not applicable.

Patient ID	Stage	Recurrence	Hotspot Mutations	gDNA Availability		Plasma cfDNA Amount (ng)			
				Tumor	Normal	Diagnosis	Postoperative	Follow-Up	
UU001	III	Yes	*KRAS* p.G13D + *PIK3CA* p.E542K	Yes	Yes	35.7	N/A	27.0	18.0
UU002	III	No	*KRAS* p.G12D	Yes	Yes	32.7	N/A	16.2	12.3
UU003	III	No	*KRAS* p.G12V + *PIK3CA* p.Q545K	Yes	Yes	12.8	N/A	8.4	12.6
UU004	IV	Yes	*KRAS* p.G12V	No	No	15.3	8.18	9.83	N/A
UU005	III	Yes	Wild-type	Yes	Yes	24.9	17.4	41.7	N/A
UU006	III	Yes	Wild-type	No	No	20.2	N/A	24.0	43.7
UU007	III	Yes	Wild-type	Yes	Yes	15.9	N/A	12.3	Leaky Tube
UU008	II	No	*KRAS* p.A146V	Yes	Yes	16.5	9.83	18.3	N/A
UU009	III	No	*BRAF* p.V600E	Yes	Yes	19.3	N/A	10.5	17.7
UU010	III	No	*KRAS* p.G12D	Yes	Yes	29.0	N/A	13.2	30.6
UU011	III	No	*KRAS* p.G12E	Yes	Yes	29.0	16.8	10.5	N/A
UU012	III	No	*BRAF* p.V600E	Yes	Yes	38.7	N/A	11.6	21.3
UU013	II	No	Wild-type	Yes	Yes	16.5	N/A	21.3	28.2
UU014	III	No	*BRAF* p.V600E	Yes	Yes	21.3	11.5	21.6	N/A
UU015	II	No	*BRAF* p.V600E + *PIK3CA* p.H1047R	Yes	Yes	18.5	N/A	27.0	12.0
UU016	I	No	Wild-type	Yes	Yes	15.5	N/A	30.9	20.4
UU017	II	No	*BRAF* p.V600E	Yes	Yes	25.3	N/A	50.1	37.8
UU018	I	No	*KRAS* p.G13D	Yes	Yes	19.6	N/A	13.8	23.4
UU019	II	No	Wild-type	Yes	Yes	11.1	12.6	7.13	N/A
UU020	I	No	*KRAS* p.G12D	No	No	32.1	N/A	20.4	20.7
UU025	IV	Yes	*NRAS* p.G12D	Yes	Yes	35.7	N/A	32.1	26.7
UU035	III	Yes	*KRAS* p.G12V	No	No	13.5	N/A	N/A	222.9
UU036	III	No	*BRAF* p.V600E	Yes	Yes	16.5	27.0	15.9	N/A
UU037	III	Yes	*KRAS* p.G13C + *PIK3CA* p.H1047L	No	No	N/A	24.0	18.0	23.4
UU038	III	Yes	*PIK3CA* p.E542K	Yes	Yes	22.8	N/A	27.0	30.9

**Table 2:** Days from surgery to diagnosis, recurrent disease, and sample time points. N/A, not applicable.

	Time from Surgery (Days)				
Patient ID	Diagnosis	Postoperative Plasma Sample	Follow-Up Plasma Samples		Recurrent Disease
UU001	−26	N/A	+744	+1112	+286
UU002	−5	N/A	+409	+1152	No
UU003	−22	N/A	+442	+1449	No
UU004 *	N/A	+42	+133	N/A	+130
UU005	−30	+43	+420	N/A	+414
UU006	−110	N/A	+113	+868	+382
UU007	−1	N/A	+437	+1214	+1187
UU008	−30	+60	+517	N/A	No
UU009	−37	N/A	+384	+1128	No
UU010	−14	N/A	+413	+1192	No
UU011	−41	+34	+1134	N/A	No
UU012	−40	N/A	+478	+1143	No
UU013	−57	N/A	+405	+1127	No
UU014	−51	+29	+1126	N/A	No
UU015	−37	N/A	+470	+1023	No
UU016	−13	N/A	+389	+1115	No
UU017	−48	N/A	+442	+1157	No
UU018	−42	N/A	+400	+1147	No
UU019	−47	+87	+414	N/A	No
UU020	−68	N/A	+397	+1147	No
UU025	−68	N/A	+217	+409	+422
UU035	−128	N/A	+161	N/A	+157
UU036	−103	+93	+443	N/A	No
UU037	N/A	+20	+170	+308	+101
UU038	−104	N/A	+119	+310	+289

* UU004 did not have surgery, and hence, time points are from diagnosis.

**Table 3:** Clinicopathological characteristics of the patients in the cohort. TNM, tumor–node–metastases; NOS, not otherwise specified; N/A, not applicable.

Characteristic	Cohort (%)
Sex	
Female	10 (40%)
Male	15 (60%)
Age at Diagnosis	
<75	15 (60%)
≥75	10 (40%)
TNM stage	
I	3 (12%)
II	5 (20%)
III	15 (60%)
IV	2 (8%)
Primary Location	
Colon	18 (72%)
Rectum	7 (28%)
Malignancy Grade	
High Grade	6 (24%)
Low Grade	18 (72%)
NOS	1 (4%)
Histology Subtype	
Adenocarcinoma	20 (80%)
Mucinous Adenocarcinoma	4 (16%)
NOS	1 (4%)
Recurrent Disease	
Yes	9 (36%)
No	16 (64%)
Surgery	24 (96%)
Neoadjuvant and Adjuvant Treatments	
Neoadjuvant Treatment	5 (20%)
Chemotherapy	1 (4%)
Radiotherapy	1 (4%)
Chemoradiotherapy	3 (12%)
Adjuvant Treatment	11 (44%)
Chemotherapy	11 (44%)
Radiotherapy	0
Chemoradiotherapy	0
Both Neoadjuvant and Adjuvant Treatment	2 (8%)
No Neoadjuvant or Adjuvant Treatment	11 (44%)
Palliative Treatment	6 (24%)

## Data Availability

The data presented in this study are available within the article and the Supplementary Material.

## Supplementary Materials

The following supporting information can be downloaded at: https://www.mdpi.com/article/10.3390/cancers16030549/s1, Table S1: The superRCA CRC Mutation assay target panel; Table S2: Mutant allele frequencies of hotspot mutations a priori known in tumor and normal gDNA by superRCA; Table S3: Mutant allele frequencies of hotspot mutations determined by superRCA in plasma cell-free DNA at diagnosis, postoperatively and follow-up; Figure S1: Days from surgery to diagnosis, postoperative and follow-up plasma samples for all 25 patients and days to recurrent disease for applicable patients. Figure S2: Total cell-free DNA input for plasma samples at diagnosis, postoperatively and during follow-up.
