# Supplementary material for: Sensitive and Specific Analyses of Colorectal Cancer Recurrence through Multiplex superRCA Mutation Detection in Blood Plasma
Source: Cancers (Basel). 2024 Jan 27;16(3):549. doi: 10.3390/cancers16030549 (PMC10854605; doi:10.3390/cancers16030549)
Supplement: Supplementary file 1 [file cancers-16-00549-s001.zip › cancers-2814118-supplementary.pdf]

cancers

MDPI

Article

Supplementary Materials

Emma Sandberg[1], Luís Nunes[1], Per-Henrik Edqvist[1], Lucy Mathot[1], Lei Chen[1,2], Tomas Edgren[2], Shahed Al Nassralla[1], Bengt Glimelius[1], Ulf Landegren[1,*] and Tobias Sjöblom[1,*]

[1] Science for Life Laboratory, Department of Immunology, Genetics and Pathology, Uppsala University, SE-751 85 Uppsala, Sweden; emma.sandberg@igp.uu.se (E.S.); luis.nunes@igp.uu.se (L.N.); per-henrik.edqvist@igp.uu.se (P.-H.E.); lucy.mathot@igp.uu.se (L.M.); lei.chen@raritybioscience.com (L.C.); shahed.alnassralla.9832@student.uu.se (S.A.); bengt.glimelius@igp.uu.se (B.G.)

[2] Rarity Bioscience AB, SE-752 37 Uppsala, Sweden; tomas.edgren@raritybioscience.com

* Correspondence: tobias.sjoblom@igp.uu.se (T.S.); ulf.landegren@igp.uu.se (U.L.)

Table S1. The superRCA CRC Mutation assay target panel.

Gene	Amplicon ID	Mutation
KRAS	KRAS12	p.G12E (c.35_56delinsAG)
		p.G12C (c.34G>T)
		p.G12R (c.34G>C)
		p.G12S (c.34G>A)
		p.G12A (c.35G>C)
		p.G12D (c.35G>A)
		p.G12V (c.35G>T)
		p.G13C (c.37G>T)
		p.G13R (c.37G>C)
		p.G13D (c.38G>A)
	KRAS61	p.A59T (c.175G>A)
		p.Q61L (c.182A>T)
		p.Q61H (c.183A>T)
		p.Q61H (c.183A>C)
	KRAS146	p.A146T (c.436G>A)
		p.K147Q (c.439A>C)
		p.A146V (c.437C>T)
NRAS	NRAS12	p.G12C (c.34G>T)
		p.G12R (c.34G>C)
		p.G12S (c.34G>A)
		p.G12A (c.35G>C)
		p.G12D (c.35G>A)
		p.G12V (c.35G>T)
		p.G13R (c.37G>C)
		p.G13D (c.38G>A)
		p.G13V (c.38G>T)
	NRAS61	p.A59T (c.175G>A)
		p.Q61K (c.181C>A)
		p.Q61R (c.182A>G
		p.Q61L (c.182A>T)
		p.Q61H (c.183A>C)
		p.Q61H (c.183A>T)
PIK3CA	PIK3CA112	p.I112F (c.334A>T)
		p.K111E (c.331A>G)

	PIK3CA545	p.E542K (c.1624G>A)<br>p.E545K (c.1633G>A)<br>p.E545G (c.1634A>G)<br>p.Q546K (c.1636C>A)
	PIK3CA1047	p.M1043I (c.3129G>T)<br>p.H1047R (c.3140A>G)<br>p.H1047L (c.3140A>T)
BRAF	BRAF600	p.V600E (c.1799T>A)<br>p.V600K (c.1798_1799 GT>AA)
AKT1	AKT1	p.E17K (c.49G>A)

Table S2. Mutant allele frequencies of hotspot mutations *a priori* known in tumor and normal gDNA by superRCA. gDNA, genomic DNA; MAF, mutant allele frequencies.

Patient ID	Hotspot Mutation	Tumor gDNA MAF	Normal gDNA MAF
UU001	*KRAS* p.G13D	0.4486	1.950×10^{-6}
	PIK3CA p.E542K	0.1314	6.498×10^{-6}
UU002	*KRAS* p.G12D	0.4869	3.855×10^{-5}
UU003	*KRAS* p.G12V	0.5072	7.978×10^{-5}
	PIK3CA p.E545K	0.0001	3.511×10^{-6}
UU008	*KRAS* p.A146V	0.3145	6.895×10^{-6}
UU009	*BRAF* p.V600E	0.2633	7.633×10^{-6}
UU010	*KRAS* p.G12D	0.2548	7.806×10^{-5}
UU011	*KRAS* p.G12E	0.3652	2.869×10^{-6}
UU012	*BRAF* p.V600E	0.1522	1.062×10^{-5}
UU014	*BRAF* p.V600E	0.1303	5.224×10^{-5}
UU015	*BRAF* p.V600E	0.2604	5.753×10^{-5}
	PIK3CA p.H1047R	0.0025	6.560×10^{-5}
UU017	*BRAF* p.V600E	0.2741	6.925×10^{-6}
UU018	*KRAS* p.G13D	0.3779	8.641×10^{-5}
UU025	*NRAS* p.G12D	0.2927	3.279×10^{-5}
UU036	*BRAF* p.V600E	0.4539	2.089×10^{-4}
UU038	*PIK3CA* p.E542K	0.0003	3.858×10^{-5}

Table S3. Mutant allele frequencies of hotspot mutations determined by superRCA in plasma cell-free DNA at diagnosis, postoperatively and follow-up. MAF, mutant allele frequencies. N/A; Not Applicable.

		Diagnosis Plasma		Postoperative Plasma		Follow-up Plasma			
**Patient ID**	**Hotspot Mutations**	**DNA input (ng)**	**MAF**	**DNA input (ng)**	**MAF**	**DNA input (ng)**	**MAF**	**DNA input (ng)**	**MAF**
UU001	*KRAS* p.G13D	35.69	1.5541×10^{-2}	N/A	N/A	27.00	9.652435×10^{-2}	18.00	1.4992918×10^{-1}
UU001	*PIK3CA* p.E542K	35.69	2.865×10^{-3}	N/A	N/A	27.00	2.17358×10^{-2}	18.00	5.616191×10^{-2}
UU002	*KRAS* p.G12D	32.72	5.040×10^{-3}	N/A	N/A	16.20	2.9701×10^{-6}	12.30	6.1279×10^{-6}
UU003	*KRAS* p.G12V	12.83	1.2092×10^{-2}	N/A	N/A	8.38	5.4617×10^{-6}	12.60	5.7408×10^{-6}
UU003	*PIK3CA* p.E545K	12.83	1.1×10^{-5}	N/A	N/A	8.38	2.8563×10^{-6}	12.60	3.2439×10^{-6}
UU004	*KRAS* p.G12V	15.26	1.680×10^{-3}	8.18	7.44464×10^{-3}	9.83	6.80364×10^{-3}	N/A	N/A
UU008	*KRAS* p.A146V	16.52	1.456×10^{-3}	9.83	1.1264×10^{-5}	18.30	6.758×10^{-6}	N/A	N/A
UU009	*BRAF* p.V600E	19.31	3.4561×10^{-2}	N/A	N/A	10.50	3.0716×10^{-6}	17.70	1.62×10^{-5}
UU010	*KRAS* p.G12D	29.03	2.930×10^{-3}	N/A	N/A	13.20	1.3208×10^{-5}	30.60	4.5509×10^{-6}
UU011	*KRAS* p.G12E	29.03	1.551×10^{-3}	16.80	1.911×10^{-6}	10.50	4.5968×10^{-6}	N/A	N/A
UU012	*BRAF* p.V600E	38.70	1.725×10^{-3}	N/A	N/A	11.64	3.821×10^{-6}	21.30	7.1307×10^{-6}
UU014	*BRAF* p.V600E	21.29	2.032×10^{-3}	11.48	4.7223×10^{-6}	21.60	5.794×10^{-6}	N/A	N/A
UU015	*BRAF* p.V600E	18.45	1.4×10^{-5}	N/A	N/A	27.00	6.9854×10^{-6}	12.00	5.4248×10^{-6}
UU015	*PIK3CA* p.H1047R	18.45	1.830×10^{-3}	N/A	N/A	27.00	5.4652×10^{-6}	12.00	9.0385×10^{-6}
UU017	*BRAF* p.V600E	25.25	3.7850×10^{-2}	N/A	N/A	50.10	7.6084×10^{-6}	37.80	4.0263×10^{-6}
UU018	*KRAS* p.G13D	19.58	5.532×10^{-3}	N/A	N/A	13.80	2.8137×10^{-6}	23.40	3.4004×10^{-6}
UU020	*KRAS* p.G12D	32.09	3.60×10^{-4}	N/A	N/A	20.40	1.0193×10^{-5}	20.70	2.2713×10^{-6}
UU025	*NRAS* p.G12D	35.70	1.136×10^{-3}	N/A	N/A	32.10	3.9785×10^{-6}	26.70	4.1889×10^{-6}
UU035	*KRAS* p.G12V	13.50	4.689×10^{-3}	N/A	N/A	33.0	3.0094103×10^{-1}	N/A	N/A
UU036	*BRAF* p.V600E	16.50	5.80×10^{-4}	27.00	1.0387×10^{-5}	15.90	3.738×10^{-6}	N/A	N/A
UU037	*KRAS* p.G13C	N/A	N/A	24.00	1.0×10^{-5}	18.00	9.7298×10^{-4}	23.40	4.460193×10^{-2}
UU037	*PIK3CA* p.H1047L	N/A	N/A	24.00	3.0×10^{-6}	18.00	1.4937×10^{-3}	23.40	2.544395×10^{-2}
UU038	*PIK3CA* p.E542K	22.80	7.0×10^{-6}	N/A	N/A	27.00	6.8573×10^{-6}	30.90	7.877×10^{-6}

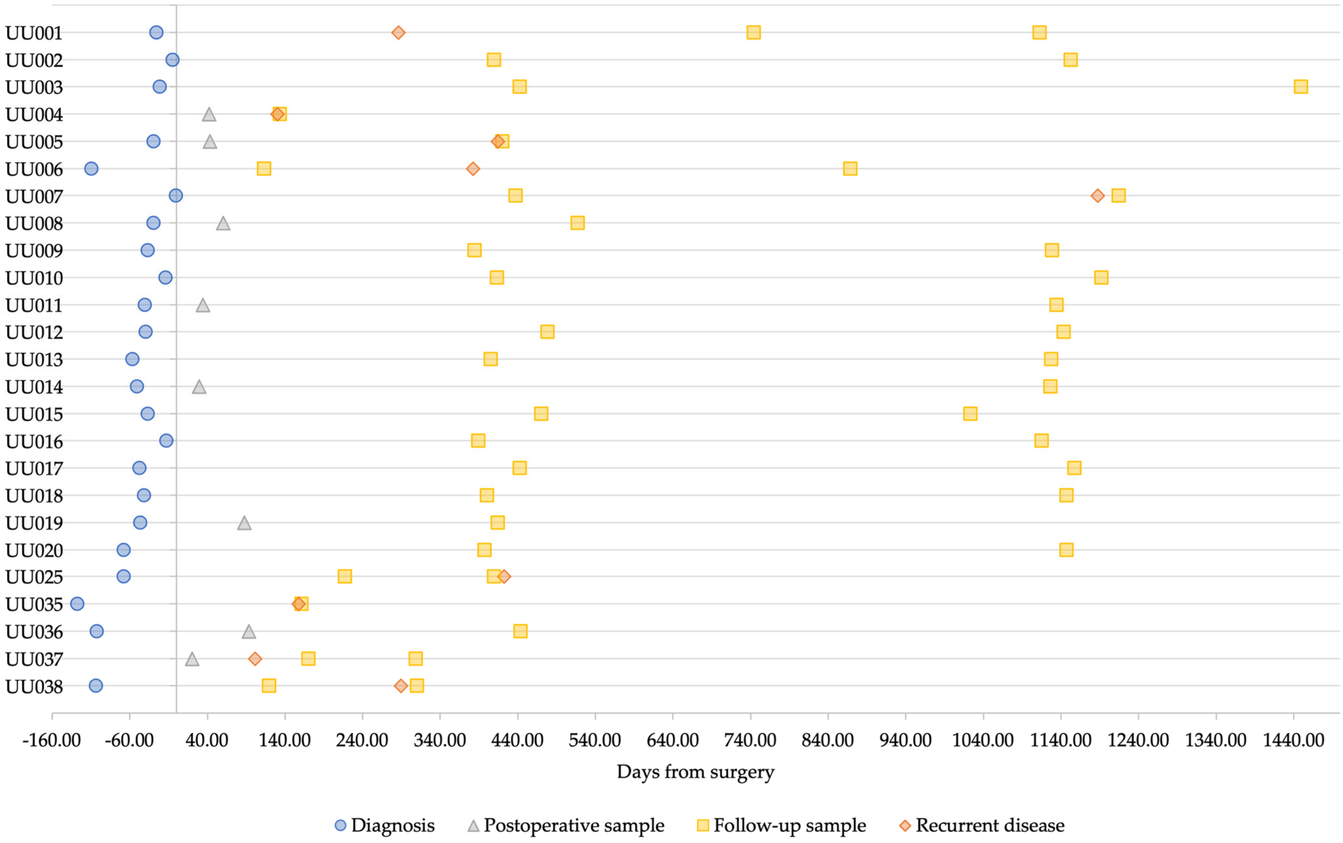

Figure S1. Days from surgery to diagnosis, postoperative and follow-up plasma samples for all 25 patients and days to recurrent disease for applicable patients. UU004 did not have surgery, hence time points are from diagnosis.

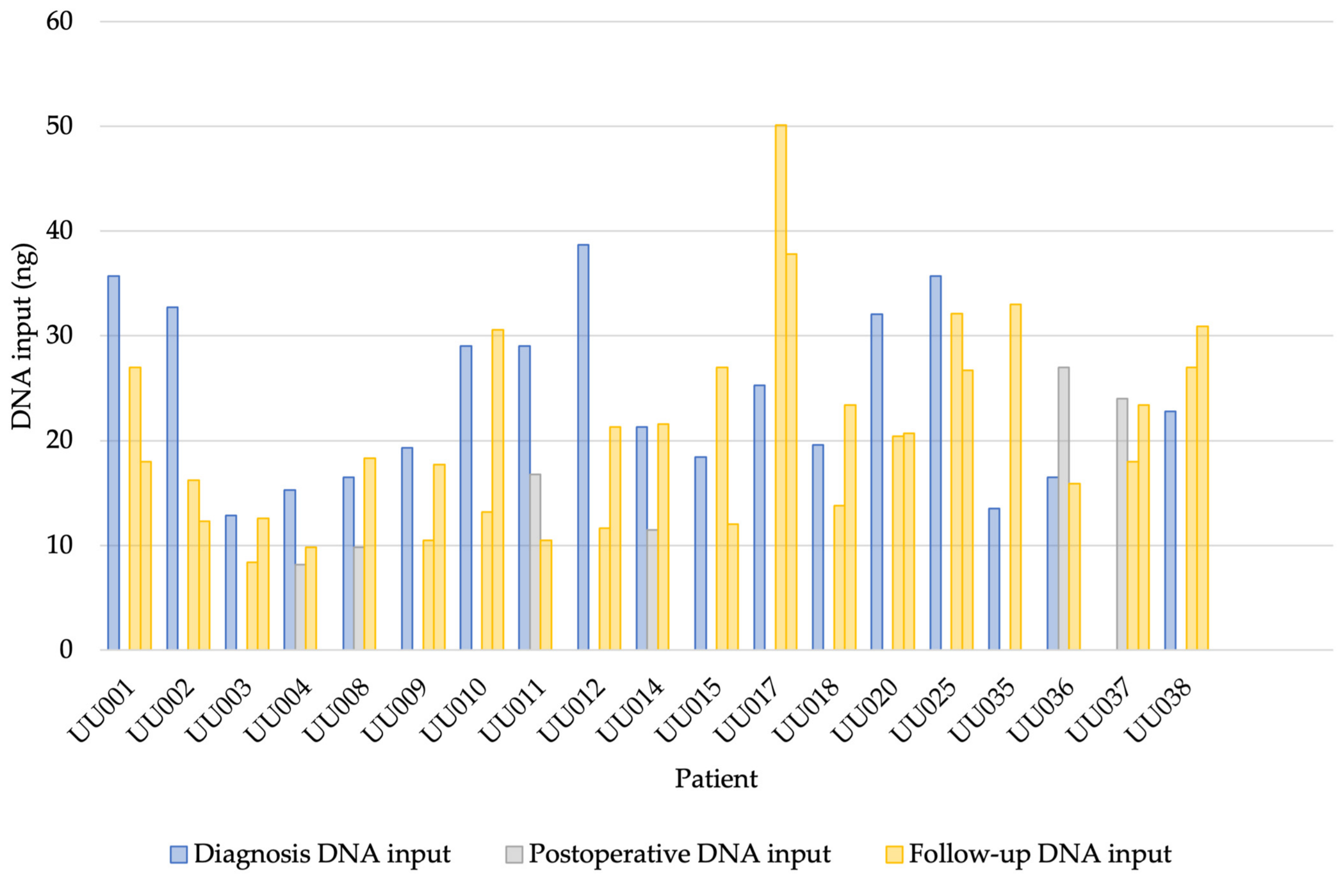

Figure S2. Total cell-free DNA input for plasma samples at diagnosis, postoperatively and during follow-up.
